# Automatic Guidance Method for Laser Tracker Based on Rotary-Laser Scanning Angle Measurement

**DOI:** 10.3390/s20154168

**Published:** 2020-07-27

**Authors:** Linghui Yang, Yuanlin Pan, Jiarui Lin, Yang Liu, Yue Shang, Shuo Yang, Hanwen Cao

**Affiliations:** State Key Laboratory of Precision Measuring Technology and Instruments, School of Precision Instruments and Optoelectronics Engineering, Tianjin University, Tianjin 300072, China; icelinker@tju.edu.cn (L.Y.); panyuanlin@tju.edu.cn (Y.P.); liuyangly@tju.edu.cn (Y.L.); ttabbx@tju.edu.cn (Y.S.); yangshuo@tju.edu.cn (S.Y.); caohw@tju.edu.cn (H.C.)

**Keywords:** automatic guidance, rotary-laser scanning, laser tracker

## Abstract

Laser-tracking measurement systems (laser tracker) have been playing a critical role in large-scale 3D high-precision coordinate measurement. However, the existing visual guidance of laser trackers is still limited by the disadvantages of operator-dependence, small-angle view field, time-consuming laser-guided process. This paper presents an automatic guidance method for laser trackers based on the rotary-laser scanning angle measurement technology. In this method, a special target consisting of six photoelectric receivers and a retroreflector is integrated into the rotary-laser scanning transmitter’ coordinate systems. Real-time constraints calculated by the proposed method would provide the coordinates of the target in a laser tracker coordinates system for guidance. Finally, the experimental results verified the automatic re-establish of sightline can be realized in horizontal 360° angle field within tens of arc-seconds, and this method is robust against the fast movement of the target.

## 1. Introduction

Benefiting from the outstanding advantages of high-precision, long-range, portable, and simple operation, laser trackers are widely used in many industrial fields such as aircraft manufacturing [1], satellite assembly [2], and large-scale gas turbine building [3]. Although it has extremely high accuracy, only one retroreflector can be tracked and measured by the principles of laser InterFerence distance Measurement (IFM) and spherical coordinate systems of laser trackers. By the IFM mode, the optical path between laser tracker and retroreflector is not allowed to be blocked during coordinate measurement and limited within its line of sight. Two solutions are generally utilized facing towards the multi-target measurement tasks in fuselage assembly or robots’ guidance, for example. One is switching tracking laser by moving and placing retroreflectors manually between multiple targets, and the other is constructing a multi-tracker network by extra laser trackers. However, the shortcoming is obviously its low efficiency from manual operation, complex calibration, and increasing cost [4,5]. Therefore, it has been a long-term concern for researchers to search for an affordable automatic detection and guidance method for multiple retroreflectors to improve the laser tracker’s multi-target measurement capability in the field of large-scale coordinates measurement.

Recently, the development of the Absolute Distance Measurement (ADM) system reduces the tracking requirement on continuous traction of retroreflectors and provides a favorable condition to realize automatic measurement. Based on this, visual methods including monocular vision measurement and binocular vision measurement have brought new approaches for target recognition and automatic guidance by integrating a single camera in the tracking hand with Leica’s PowerLock, for example. The camera captures the images and rotates with the tracker head, and the coordinates of its center under the camera coordinate system can be obtained using the reflector’s geometric features once a retroreflector is detected in the captured image. The pointing direction of the measuring laser can be calculated by coordinate transformation, and the tracking head is guided to the reflector precisely and automatically. If the laser beam was temporarily blocked, tracking and measuring can be restored automatically in a Field of View (FOV) of ±5° [6,7]. Compared with the monocular camera, the binocular stereo camera can measure the coordinates of specific targets directly, and the requirement for target recognition is reduced while FOV is enlarged. Typical binocular vision measurement as Active Seek stereo camera using in FARO’s laser tracker can get a 50° FOV.

Although visual-measurement-based guidance is currently most studied, many problems are still encountered in application fields [8,9,10,11]. Firstly, limited by the FOV of the camera lens, the vision-based guidance method can only sense and guide the retroreflector within a certain angle range. When faced with large-scale application scenarios such as on-site aircraft fuselage measurement, the FOV of a camera lens still cannot cover all targets. Retroreflectors must be manually taught to switch between multiple measuring points without initial pointing input. Secondly, due to the limited number of camera pixels, the farther the measuring target is, the less effective pixels it takes in an image, and the more difficult it is to perform feature extraction. Under a complex background of a real scene, for small-sized targets such as 0.5-inch reflector or light spots on measuring probe, the current vision-based guidance is still not satisfied when the working range is extended to more than 10 m. In addition, due to the camera’s inherent resolution–range contradiction, the target recognition efficiency in complex scenes is difficult to increase together with the camera resolution. The higher the resolution is, the longer the required time for image transmission, storage, and target recognition is. Thus, dynamic tracking is still a difficult problem for visual guidance systems. Therefore, a new guidance method with a large field of view, long distance, and automatic detection capability is extremely valuable for the field application of laser trackers.

Presently, in addition to the vision-based guidance method, tracking and positioning technology based on rotary-laser scanning is also attracting widespread attention in large-scale spatial coordinate measurement research [12,13]. Based on the principle of Angle of Arrival (AoA), rotary-laser scanning systems usually achieve orientation or positioning by cooperating with photoelectric receivers. Receivers are placed at a far end to detect the spatial scanning optical signals generated by rotating mechanism. Without the restrictions on image resolution and hardware processing, rotary-laser scanning systems were usually featured with fast scanning speed, longer working distance, and higher spatial resolution. It has been successfully demonstrated in several high-efficiency equipments, such as the Structural Health Monitoring system (SHM) [14,15], HTC Vive [16,17,18], indoor Global Position System (iGPS) [19,20], and workspace Measuring and Positioning System (wMPS) [21,22,23,24]. SHM makes full use of rotary-laser scanning technology to determine three-dimensional (3D) coordinates of an observed object efficiently. The HTC Vive consists of two controllers, a headset, and a Lighthouse, which could alternatingly send out horizontal and vertical infrared laser planes with 120-degree sweeps spanning in each direction. By scanning multi-markers, the system can track handles and the headset with submillimetre accuracy and 90 Hz refresh rate in a room-scale space of 8 m [16]. iGPS and wMPS adopt a similar working principle with farther measurement distance, and they support multiple targets measurement. They both consist of a set of rotary-laser transmitter and photoelectric receivers. Each transmitter emits two rotating laser planes with different tilt angles and a synchronized light pulse. The receiver takes charge of the photoelectric conversion of transmitters’ optical signals and sends them to an embedded processor to record the rotary time. Receivers’ coordinates can be calculated in real time from the multi-transmitters optical signals based on the triangulation principle. Taking the wMPS system, for example, the working distance of a single transmitter is up to 25 m, with a scanning coverage range of 360° horizontal angles and ±22.5° vertical angles. It supports multi-targets measurement with measuring frequency up to 30 Hz [23,24]. Compared with visual measurement, it has significant advantages in range, accuracy, and efficiency, so that it is more suitable for on-site positioning and guidance.

In this paper, we proposed a novel automatic guidance method for laser trackers based on the technology of rotary-laser scanning. A target combining photoelectric receiver and retroreflector was designed to unify the coordinates system between transmitter and laser tracker for achieving automatic guidance. The coordinate fusion method between the tracker and the transmitter is proposed, and the simulation analysis and experimental verification are carried out. The experimental results show that our method can sense measuring targets within 360° field and guide the laser tracker automatically with high measurement efficiency. The rest of this paper is organized as follows. Principles of laser tracker and wMPS are given in Section 2; automatic guidance principle, calibration method of intrinsic and extrinsic parameters, and system error analysis are described in Section 3; in Section 4, the effectiveness and the robustness of the proposed automatic guidance method are verified by repeated site-guided experiments and trajectory experiments. Finally, the conclusion and the work in the future are analyzed in Section 5.

## 2. Principles and Methods

Fundamental and structural characteristic introductions on laser trackers and wMPS are conducted in this section, including the reason why laser trackers need an automatic guidance method, and the working principle of wMPS, which shows the multi-point real-time searching ability in horizontal full angles. Coordinate system definitions of the two main equipment prepare for the guidance principle and experiments description.

### 2.1. Laser-Tracker

**Figure 1:**
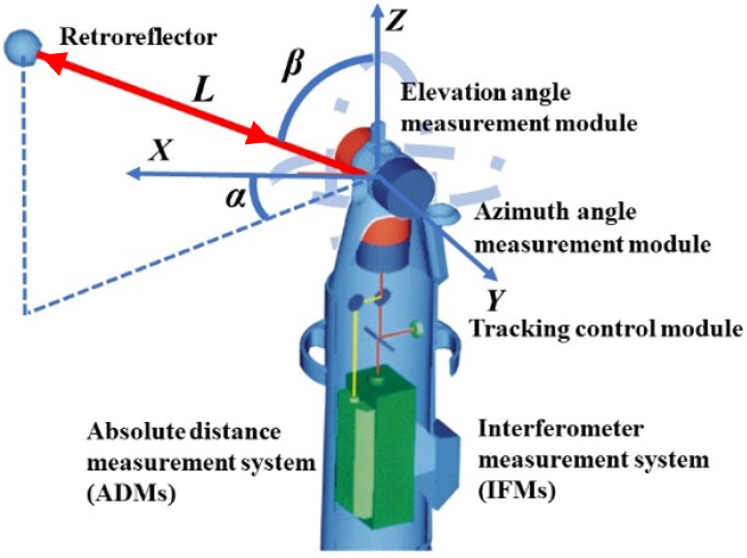
The typical coordinate system of a laser tracker.

As shown in Figure 1, laser tracker is mainly composed of retroreflector and several function modules, including the distance measurement module, angle-measuring module, and tracking control module [25,26,27]. The distance measurement module includes IFMs and ADMs.

The angle measurement module consists of two mutually perpendicular angle encoders, which are responsible for measuring and recording horizontal and vertical angles of the laser beam from coordinate origin to target point. A laser beam is emitted from the tracker and reflected by the retroreflector. The reflected laser is detected by PSD (Positioning Sensitive Detector) of the tracking control module to analyze the distance between laser tracker and the retroreflector. Then, the data from PSD as feedback is sent to the servo system, which will guide the laser beam to track retroreflector continually. The laser tracker loses light constantly when the retroreflector moves too fast or the span is too broad. ADMs of laser trackers support the discontinuous guidance method.

From the principles and structures introduction above, laser trackers are designed based on the spherical coordinate system, which determines that only one retroreflector can be tracked and measured by laser tracker at the same time. A multi-point efficiency guidance method is needed. As shown in Figure 1, the Z-axis of the spherical laser-tracker coordinate system is the rotation axis of the horizontal encoder, the X-axis is the rotation axis of the vertical encoder, and the Y-axis is determined with the right-hand rule. The calculating process of the target coordinates is presented in Equation (1).

(1) $$\left\{ \begin{array}{l} X=L\cos\beta \cos\alpha \\ Y=L\cos\beta \sin\alpha \\ Z=L\sin\beta \end{array}\right.$$

### 2.2. Rotary-Laser Scanning Angle Measurement

Rotary-laser scanning systems commonly consist of several transmitters and plenty of photoelectric receivers. Two laser planes with different tilt angles are emitted by each transmitter, and receivers capture the optical signals and record the rotary time of each laser plane. While working, synchronous laser pulse is emitted from the transmitter when the transmitter’s rotating head passes through its initial position, receiver *i* (*i* = 1, 2, 3, 4……) records a start time of rotating $t_{0}$. Two end signals are sent when the two laser planes reach the target receiver, respectively, and corresponding times $t_{1i}$ and $t_{2i}$ are recorded by receivers. Therefore, rotating angles $\theta_{1}^{t_{1i}}$ and $\theta_{2}^{t_{2i}}$ could be obtained according to calculation. Rotation rates of transmitters differ from each other, generally more than 1650 r/min [28,29]. The scanning laser planes could cover a 360° wide space and construct a light field to provide angular signals for receivers. Receivers capture the rotary-laser scanning signals automatically when they are placed in the workspace. Signals from multiple transmitters can be distinguished and received simultaneously according to transmitters’ different rotation rates. Therefore, the number of transmitters can be increased or decreased according to detailed requirements. In the large-scale manufacturing field, movement of target points of laser trackers is usually slow, while the scanning period of transmitters is usually about tens of arc-seconds; therefore, the measurement conducted by the transmitter can be regarded as real-time detection. The rotary-laser scanning guidance technology shows the potential of enlarging the automatic guidance view field of laser tracker from 10 degrees to 360 degrees [30].

**Figure 2:**
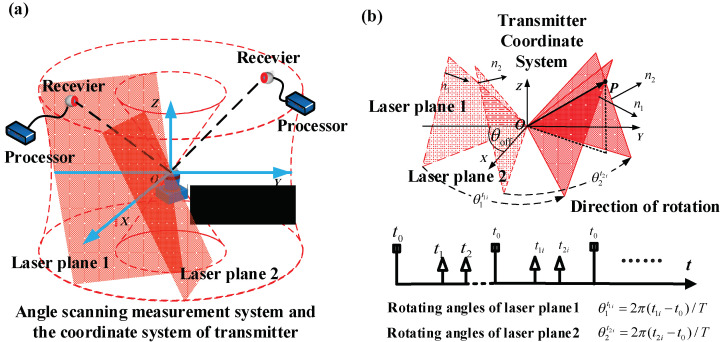
Rotary-laser scanning transmitter system: (**a**) transmitter coordinate system and angle scanning measurement system: transmitter, receiver, two light planes with fixed dip angle emitted by the transmitter; (**b**) schematic diagram of angle measurement.

The coordinate system of rotary-laser scanning transmitter (the SCS) O-XYZ is defined as follows. The rotation axis of the two light planes serves as Z-axis of the SCS. Origin O is the intersection of laser plane 1 and Z-axis. In Figure 2, the two light planes from the transmitter are marked as “laser plane 1” and “laser plan 2” for distinction. The X-axis passes through the origin O and lies in laser plane 1 at th initial position and perpendicular to Z-axis; the Y-axis is determined according to the right-hand rule.

## 3. Method of Automatic Guidance

The novel automatic guidance method for laser trackers proposed in this paper suits all laser trackers with ADMs. The laser tracker used in this paper is Leica AT901, with a measurement accuracy of 15 μm/m ± 6 μm/m in total measurement range. We design a cooperative target for guidance experiment, which is introduced in Section 3.1. In Section 3.2, the guidance method is elaborated completely. The internal and external parameters calibration methods of the system are represented in Section 3.3. Mathematical analysis of system errors is introduced in Section 3.4.

### 3.1. Cooperative Target for Guidance

Due to the differences in mechanical structure and sensing principles between transmitter and laser tracker, their optical origins cannot be aligned physically and precisely. A specially designed target is necessary for establishing the connection between retroreflector and receivers, which is important for the transmitter to get the retroreflector coordinates in SCS. Therefore, we draw on the principle of single-station positioning in the field measurement of the laser transmitter and further establish the following constraint relationship.

If the number of receivers is n in the workspace, 2*n* plane constraint equations could be established. In the condition that coordinates of receivers in TCS are fixed, there are $C_{n}^{2}$ distance equations that can be gotten from the target.

(2) $$l_{ij}=\sqrt{{\left(x_{i}-x_{j}\right)}^{2}+{\left(y_{i}-y_{j}\right)}^{2}+{\left(z_{i}-z_{j}\right)}^{2}}\;(1\leq j\leq i\leq 6)$$

(3) $$C_{n}^{2}=\frac{n(n-1)}{2},\;N=2n+C_{n}^{2}$$

where, $l_{ij}$ is the distance between them, $c_{n}^{2}$ is the number of distance constraints among receivers, 2*n* is the number of light plane constraints, ($x_{i}$, $y_{i}$, $z_{i}$) and ($x_{j}$, $y_{j}$, $z_{j}$) are coordinates of *i*-receiver and *j*-receiver, respectively, in the TCS, and N is the number of total constraints.

The conversion matrix between TCS and SCS, 3 rotation angle parameters and 3 translation parameters, can be determined through enough constraints. The four-element method is usually used to express transformation relationship of coordinate system. Rotation matrix and translation matrix between TCS and SCS contain 12 unknown parameters. Therefore, the determination of the coordinates of retroreflector turns into seeking transformation matrix. From Equation (3), the number of equations is greater than 14 when n≥4. The 12 unknown parameters could be calculated. However, in view of the target volume and the coplanar points, the target in this paper is designed with 6 receivers [30].

**Figure 3:**
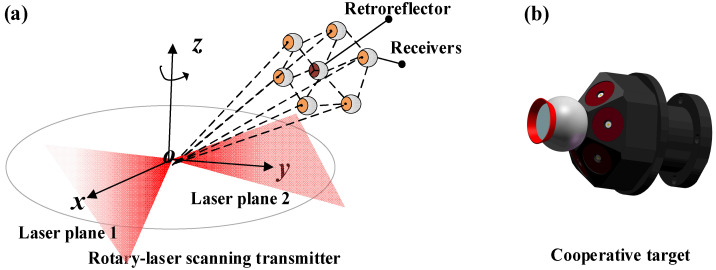
Measurement principle of single-station rotary-laser transmitter: (**a**) six receivers and a retroreflector with fixed position relationship; (**b**) cooperative target designed based on the principle of single station measurement and applied to guidance experiment in this paper.

The target, shown in Figure 3b, is designed as a regular hexahedron with six receivers, and retroreflector of the laser tracker is placed at the center of the bottom surface. It can make full use of the small area to achieve the largest possible constraints by 3D design. In the experiment, it is mounted on a tripod with a pulley to guide the laser beam of the laser tracker.

### 3.2. Principle of Automatic Guidance

**Figure 4:**
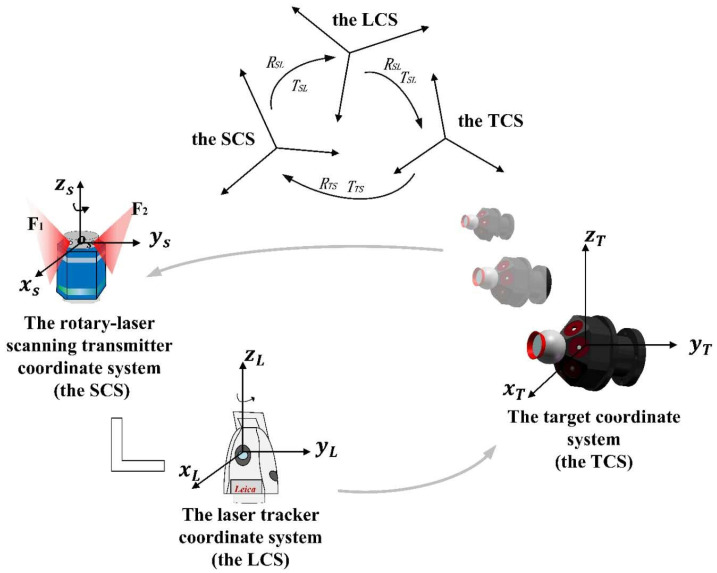
Principle of the guidance system based on rotary-laser scanning technology.

The automatic guidance system for laser tracker based on rotary-laser scanning transmitter is shown in Figure 4, where $O_{T}-X_{T}Y_{T}Z_{T}$ is the target coordinate system (TCS), $O_{R}-X_{R}Y_{R}Z_{R}$ is the coordinate system of rotary-laser scanning transmitter (SCS), and $O_{L}-X_{L}Y_{L}Z_{L}$ is the laser tracker coordinate system (LCS). R and T are rotation matrix and translation matrix, respectively.

Six receivers and a retroreflector with the fixed position are placed on the target in our experiment, the target is shown in Figure 4. Two light planes start from the initial position and pass through each receiver in turn during rotation.

Light plane equations in the initial position are,

(4) $$\begin{array}{l} F_{1}=a_{1}^{t_{0}}x+b_{1}^{t_{0}}y+c_{1}^{t_{0}}z\\ F_{2}=a_{2}^{t_{0}}x+b_{2}^{t_{0}}y+c_{2}^{t_{0}}(z+\Delta z) \end{array}$$

where the $a_{m}^{t_{0}}$,$\;b_{m}^{t_{0}}$,$\;c_{m}^{t_{0}}$ (*m* = 1, 2) are the plane coefficients of the *m*-light plane at the initial position, ∆z is the intercept of light plane 2 on Z-axis, and $F_{1}$ and $F_{2}$ are the equations of the two light planes, which has been pointed out in Figure 4.

As shown in Figure 2, when a scanning light plane reaches receivers, the plane equations could be calculated by the recorded rotary time from the processor.

(5) $$\theta_{m}^{t_{mi}}=\omega (t_{mi}-t_{0})(m=1,2)$$

where the $\theta_{m}^{t_{mi}}$ is the rotary-angle of *m*-light plane from initial position to *i*-receiver.

The laser planes transformation matrix can be calculated based on the time detection by the receiver. Then twelve light plane constraints can be obtained. The detail process is as follows,

(6) $$\left[\begin{array}{c} a_{m}^{t_{mi}}\\ b_{m}^{t_{mi}}\\ c_{m}^{t_{mi}}\\ d_{m}^{t_{mi}} \end{array}\right]=\left[\begin{array}{cccc} \cos\theta_{m}^{t_{mi}} & -\sin\theta_{m}^{t_{mi}} & 0 & 0\\ \sin\theta_{m}^{t_{mi}} & \cos\theta_{m}^{t_{mi}} & 0 & 0\\ 0 & 0 & 1 & 0\\ 0 & 0 & 0 & 1 \end{array}\right]\left[\begin{array}{c} a_{m}^{t_{0}}\\ b_{m}^{t_{0}}\\ c_{m}^{t_{0}}\\ d_{m}^{t_{0}} \end{array}\right],m=1,2$$

where the $\theta_{m}^{t_{mi}}$ is the angle of the *m*-light plane from the initial position to the *i*-receiver. The $a_{m}^{t_{mi}}$,$\;b_{m}^{t_{mi}}$,$\;c_{m}^{t_{mi}}$ (*m* = 1, 2) are the coefficients of *m*-light plane when reaching the *i*-receiver, and $\;a_{m}^{t_{0}}$,$\;b_{m}^{0}$,$\;c_{m}^{t_{0}}$ (*m* = 1, 2) is the coefficients of *m*-light plane in the initial position.

Converting coordinates of receivers from TCS to SCS,

(7) $$\left[\begin{array}{c} x\\ y\\ z\\ 1 \end{array}\right]=\left[\begin{array}{cc} R_{TS} & T_{TS}\\ 0 & 1 \end{array}\right]\left[\begin{array}{c} x_{0}\\ y_{0}\\ z_{0}\\ 1 \end{array}\right]=\left[\begin{array}{cccc} r_{1} & r_{2} & r_{3} & t_{x}\\ r_{4} & r_{5} & r_{6} & t_{y}\\ r_{7} & r_{8} & r_{9} & t_{z}\\ 0 & 0 & 0 & 1 \end{array}\right]\left[\begin{array}{c} x_{0}\\ y_{0}\\ z_{0}\\ 1 \end{array}\right]=\left[\begin{array}{c} r_{1}x_{0}+r_{2}y_{0}+r_{3}z_{0}+t_{x}\\ r_{4}x_{0}+r_{5}y_{0}+r_{6}z_{0}+t_{y}\\ r_{7}x_{0}+r_{8}y_{0}+r_{9}z_{0}+t_{z}\\ 1 \end{array}\right]$$

where the *x*, *y*, *z* are coordinates of the receiver in SCS, and $x_{0},y_{0},z_{0}$ are coordinates of the receiver in TCS. $R_{TS}$ and $T_{TS}$ are rotation matrix and translation matrix between TCS and SCS.

Creating light plane equations based on coordinates of a point in SCS,

(8) $$\begin{array}{l} F_{1}^{i}=(a_{1}^{t_{0}}\cos\theta_{1}^{t_{1i}}-b_{1}^{t_{0}}\sin\theta_{1}^{t_{1i}})x+(a_{1}^{t_{0}}\sin\theta_{1}^{t_{1i}}+b_{1}^{t_{0}}\cos\theta_{1}^{t_{1i}})y+c_{1}^{t_{0}}z\\ F_{2}^{i}=(a_{2}^{t_{0}}\cos\theta_{2}^{t_{2i}}-b_{2}^{t_{0}}\sin\theta_{2}^{t_{2i}})x+(a_{2}^{t_{0}}\sin\theta_{2}^{t_{2i}}+b_{2}^{t_{0}}\cos\theta_{2}^{t_{2i}})y+c_{2}^{t_{0}}z+d_{2}^{t_{0}}\;\;\\ \;(i=1,2,3,4,5,6) \end{array}$$

where $F_{1}^{i}$ and $F_{2}^{i}$ represent the light plane equations when light plane 1 and light plane 2 reach the *i*-th receiver, respectively.

The elements of the rotation matrix $R_{TS}$, represented by the quaternion method [30,31,32], have a constraint relationship as shown below,

(9) $$\left\{ \begin{array}{l} f_{1}=r_{1}^{2}+r_{2}^{2}+r_{3}^{2}-1=0\\ f_{2}=r_{4}^{2}+r_{5}^{2}+r_{6}^{2}-1=0\\ f_{3}=r_{7}^{2}+r_{8}^{2}+r_{9}^{2}-1=0\\ f_{4}=r_{1}r_{4}+r_{2}r_{5}+r_{3}r_{6}=0\\ f_{5}=r_{1}r_{7}+r_{2}r_{8}+r_{3}r_{9}=0\\ f_{6}=r_{4}r_{7}+r_{5}r_{8}+r_{6}r_{9}=0 \end{array}\right.$$

where $r_{i}$ (*i* = 1, 2……9) are the coefficients expressed by the four-element method of the rotation matrix.

(10) $$F=\sum_{i=1}^{6}\left((F_{1}^{i}\right){}^{2}+(F_{2}^{i}){}^{2}+\sum_{j=1}^{6}f_{j}^{2}$$

where *F* is the optimization equation established by multiple constraints.

$R_{TS}$ and $T_{TS}$ can be calculated through the optimized function described in Equation (10). $R_{SL}$ and $T_{SL}$, treated as system parameters, could be calculated previously by high-precision 3D control field, which is described in Section 3.3. Then, converting coordinates of retroreflector in TCS to LCS,

(11) $$X=R_{SL}(R_{TS}X_{0}+T_{TS})+T_{SL}=R_{SL}R_{TS}X_{0}+(R_{SL}T_{TS}+T_{SL})$$

where $X_{0}$ and *X* are coordinates of retroreflector in TCS and LCS, respectively. Converting Cartesian coordinates into angle value in the spherical coordinate system, the mathematical transformation is as follows [7],

(12) $$\left\{ \begin{array}{l} r=\sqrt{X_{\left(1\right)}{}^{2}+X_{\left(2\right)}{}^{2}+X_{\left(3\right)}{}^{2}}\\ \alpha =\arctan(\frac{X_{\left(2\right)}}{X_{\left(1\right)}})\\ \beta =\arccos(\frac{X_{\left(3\right)}}{r}) \end{array}\right.$$

where ($X_{\left(1\right)}$, $X_{\left(2\right)}$, $X_{\left(3\right)}$) are the Cartesian coordinates of the target point in the LCS, r represents the distance between the target and origin of the LCS, and α and β are the horizontal and elevation angles that the laser tracker needs to turn to reach the retroreflector, respectively.

Coordinates of retroreflectors in TCS are known. Coordinates of retroreflectors in SCS, $R_{TS}$ and $T_{TS}$, are calculated through the method explained in Equations (4)–(10). Equations (11) and (12) give the angle to be turned by the laser tracker for reaching retroreflector. Then, they are provided to servo system of laser tracker for laser beam instruction through the program developed based on EMSCON. Target points in measurement space can be locked automatically without view field limitation in this method. Compared with the existing positioning method, the one based on rotary-laser scanning technology greatly improves efficiency by avoiding the continuous guidance process of the laser beam.

### 3.3. System Calibration

**Figure 5:**
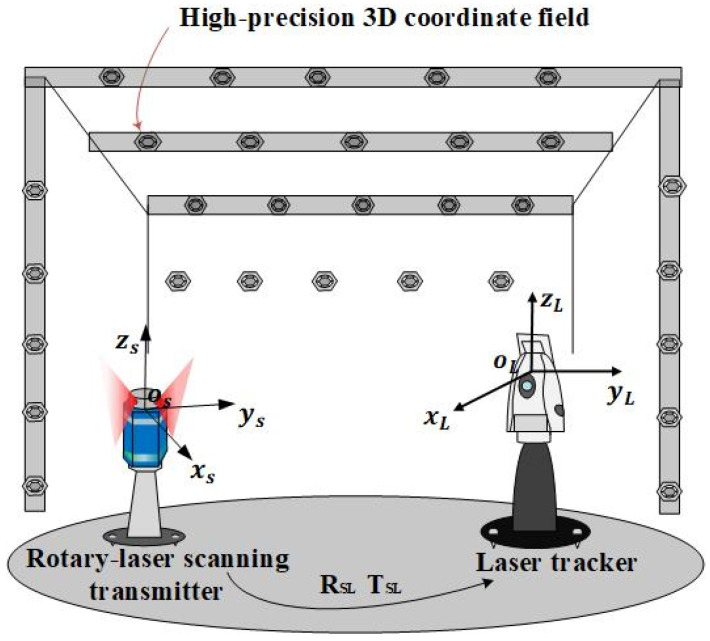
Layout of high-precision 3D control field, laser tracker, and rotary-laser scanning transmitter.

Considering that the laser tracker itself has extremely high spatial coordinate measurement accuracy, the coordinates system of laser trackers is regarded as the global coordinate system in for system calibration. We take coefficients of two laser planes at the initial position, which is emitted by the transmitter as transmitter’s eight internal parameters, and transformation matrix between LCS and SCS, $R_{SL}$ and $T_{SL}$, as transmitter’s extrinsic parameters. Both intrinsic and extrinsic parameters can be calibrated by high-precision 3D control field measured by laser tracker. As shown in Figure 5, n control points are arranged in control field.

The specific process is as follows. The high-precision 3D control field as shown in Figure 5 is established at proper position in the workspace. Coordinates of control points in the world coordinate system are calibrated before. Step 1: Establish the bridge between SCS and the world coordinate system (LCS) through control points.

(13) $$\left[\begin{array}{c} x^{’}\\ y^{’}\\ z^{’}\\ 1 \end{array}\right]=\left[\begin{array}{cc} R_{LS} & T_{LS}\\ 0 & 1 \end{array}\right]\left[\begin{array}{c} x\\ y\\ z\\ 1 \end{array}\right]=\left[\begin{array}{cccc} v_{1} & v_{2} & v_{3} & w_{1}\\ v_{4} & v_{5} & v_{6} & w_{2}\\ v_{7} & v_{8} & v_{9} & w_{3}\\ 0 & 0 & 0 & 1 \end{array}\right]\left[\begin{array}{c} x\\ y\\ z\\ 1 \end{array}\right]$$

where *x*, *y*, *z* and *x*’, *y*’, *z*’ are coordinates of control points in LCS and SCS, respectively, and $v_{1},v_{2},v_{3}\ldots \ldots v_{9,},w_{1},w_{2},w_{3}$ are parameters of $R_{LS}$ and $T_{LS}$, that is, external parameters needed to be calibrated.

Step 2: Calculating light plane coefficient by rotation time from initial position to each control point, which is measured by receivers [32].

(14) $$\left[\begin{array}{c} a_{i}^{’}\\ b_{i}^{’}\\ c_{i}^{’}\\ d_{i}^{’} \end{array}\right]=\left[\begin{array}{cccc} \cos\theta & -\sin\theta & 0 & 0\\ \sin\theta & \cos\theta & 0 & 0\\ 0 & 0 & 1 & 0\\ 0 & 0 & 0 & 1 \end{array}\right]\left[\begin{array}{c} a_{i}\\ b_{i}\\ c_{i}\\ d_{i} \end{array}\right](i=1,2)$$

where $a_{i}$,$\;b_{i}$,$\;c_{i}$,$\;d_{i}$ and $\;{a_{i}}^{’}$, $\;{b_{i}}^{’}$,$\;{d_{i}}^{’}$ are coefficients of *i*-th laser plane in initial position and when reaching control point, respectively. θ is the rotary-angle of light plane from initial position to control point.

Step 3: The coordinates of control point obtained from Equation (13) in SCS are substituted into the light plane equation.

(15) $$T=\left[\begin{array}{cccc} a^{‘}{}_{11} & b^{’}{}_{11} & c^{‘}{}_{11} & d^{’}{}_{11}\\ a^{‘}{}_{21} & b^{’}{}_{21} & c^{‘}{}_{21} & d^{’}{}_{21}\\ \vdots & \vdots & \vdots & \vdots \\ a^{‘}{}_{1n} & b^{’}{}_{1n} & c^{‘}{}_{1n} & d^{}{}_{1n}\\ a^{’}{}_{2n} & b^{‘}{}_{2n} & c^{’}{}_{2n} & d^{‘}{}_{2n} \end{array}\right]\left[\begin{array}{c} x^{‘}{}_{i}\\ y^{’}{}_{i}\\ z^{‘}{}_{i}\\ 1 \end{array}\right](i=1,2,3...30,n=30)$$

where $x_{i}^{’}$,$\;y_{i}^{’}$,$\;z_{i}^{’}$ are coordinates of the *i*-th control point in SCS, $d_{1n}$ is usually zero, $d_{2n}$ are the intersection coordinates of the two light planes and the z-axis of the transmitter respectively. $a_{mn}^{’}$, $b_{mn}^{’}$, $c_{mn}^{’}$ (*m* = 1, 2; *n* = 30) represent the plane coefficient when the m-th light plane reaches the n-th control point, and T is the matrix of the light plane equation at control points.

Step 4: In summary, when the number of control points is n in the calibration field, 2n plane equation constraints on intrinsic and extrinsic parameters can be obtained. Substituting Equations (14) and (15) into (16) gives Equation (16),

(16) $$\begin{array}{l} \left\{\begin{array}{l} T_{1i}=(a_{1}\cos\theta_{1i}-b_{1}\sin\theta_{1i})(v_{1}x_{i}+v_{2}y_{i}+v_{3}z_{3}+w_{1})+\\ (a_{1}\sin\theta_{1i}-b_{1}\cos\theta_{1i})(v_{4}x_{i}+v_{5}y_{i}+v_{6}z_{i}+w_{2})+c_{1}(v_{7}x_{i}+v_{8}y_{8}+v_{9}z_{i}+w_{3})=0\\ T_{2i}=(a_{2}\cos\theta_{2i}-b_{2}\sin\theta_{2i})(v_{1}x_{i}+v_{2}y_{i}+v_{3}z_{3}+w_{1})+\\ (a_{2}\sin\theta_{2i}-b_{2}\cos\theta_{2i})(v_{4}x_{i}+v_{5}y_{i}+v_{6}z_{i}+w_{2})+c_{1}(v_{7}x_{i}+v_{8}y_{8}+v_{9}z_{i}+w_{3})+d_{2i}=0 \end{array}\right\}\\ \left(i=1,2,3\ldots \ldots n\right) \end{array}$$

where $T_{1i}$ and $T_{2i}$ are the plane equation of the scanning light at the *i*-th control point under the coordinate system of the transmitter.

Then the calibration can be finished by the optimization function shown below,

(17) $$H=\sum_{i=1}^{n}(T_{1i}^{2}+T_{2i}^{2})+\sum_{j=1}^{6}f_{j}^{2}$$

where *H* is the optimization function equation for solving internal and external parameters. System parameters needed for positioning are obtained through the steps above. Positions of transmitter and laser tracker remain unchanged during measurement process after calibration; that is, the rotary-laser scanning system is fixed as the guidance subsystem of the laser tracker.

### 3.4. Error Analysis

In this Section, the main errors are analysis, and an error simulation experiment is designed to show guidance error changes at various locations in space. Errors in this system are the main coordinates calibration error of receivers and retroreflector in TCS and angle measurement error of the transmitter. The angle measurement error of the transmitter has been confirmed, which was below 2” [24]. The three errors would cause guidance error directly through the coordinate solutions process, as expressed in Equations (18) and (19).

Errors from target stay unchanged by calibrating. That means the guidance error deviation caused by the target will be a fixed value when the target moves. Errors from target are divided into two parts: coordinates error of retroreflector and receivers. From Equation (11), coordinates error of retroreflector affects the guidance results through Equation (18).

(18) $$\left[\begin{array}{c} ∆x_{l}\\ ∆y_{l}\\ ∆z_{l}\\ 1 \end{array}\right]=\left[\begin{array}{cc} R_{SL} & T_{SL}\\ 0 & 1 \end{array}\right]\left[\begin{array}{cc} R_{TS} & T_{ts}\\ 0 & 1 \end{array}\right]\left[\begin{array}{c} ∆x_{t}\\ ∆y_{t}\\ ∆z_{t}\\ 1 \end{array}\right]$$

where ($∆x_{t}$, $∆y_{t}$, $∆z_{t}$ ) and ($∆x_{l}$, $∆y_{l}$, $∆z_{l}$) are the calibration error of retroreflector coordinates in TCS and the guidance error caused by it, respectively; $R_{SL}$ and $T_{SL}$, are the transformation matrix between SCS and LCS; and $\;R_{TS}$ and $T_{TS}$ are the transformation matrix between TCS and SCS.

From Equations (4)–(16), coordinates calibration error of receivers will affect conversion matrix between TCS and SCS. The solution method makes this error finally reflected in the optimization equation, and the specific mathematical expression is shown in Equation (19). It is obtained by substituting receivers’ coordinates error into Equations (7) and (8).

(19) $$\left[\begin{array}{c} T_{1i}+∆T_{1i}\\ T_{2i}+∆T_{2i} \end{array}\right]=\left[\begin{array}{cccc} \begin{array}{l} a_{1i}\\ a_{2i} \end{array} & \begin{array}{l} b_{1i}\\ b_{2i} \end{array} & \begin{array}{l} c_{1i}\\ c_{2i} \end{array} & \begin{array}{l} d_{1}\\ d_{2} \end{array} \end{array}\right]\left[\begin{array}{cccc} r_{1} & r_{2} & r_{3} & t_{x}\\ r_{4} & r_{5} & r_{6} & t_{y}\\ r_{7} & r_{8} & r_{9} & t_{z}\\ 0 & 0 & 0 & 1 \end{array}\right]\left[\begin{array}{c} x_{i}+∆x_{i}\\ y_{i}+∆y_{i}\\ z_{i}+∆z_{i}\\ 1 \end{array}\right]=\left[\begin{array}{cccc} \begin{array}{l} a_{1i}\\ a_{2i} \end{array} & \begin{array}{l} b_{1i}\\ b_{2i} \end{array} & \begin{array}{l} c_{1i}\\ c_{2i} \end{array} & \begin{array}{l} d_{1}\\ d_{2} \end{array} \end{array}\right]\left[\begin{array}{c} x_{i}^{’}+∆x_{i}^{’}\\ y_{i}^{’}+∆y_{i}^{’}\\ z_{i}^{’}+∆z_{i}^{’}\\ 1 \end{array}\right]$$

where $∆T_{1i}$ and $∆T_{2i}$ are the errors from the last optimization function elements caused by coordinates error of receivers in the TCS. ($x_{i}$, $y_{i}$, $z_{i}$) are the *i*-th receiver’s coordinates in TCS, ($x_{i}{}^{\text{’}}$, $x_{i}{}^{\text{’}}$, $x_{i}{}^{\text{’}}$) are the i-th receiver’s coordinates in SCS, and ($∆x_{i}$, $∆y_{i}$, $∆z_{i}$) and ($∆x_{i}{}^{\text{’}}$, $∆y_{i}{}^{\text{’}}$, $∆z_{i}{}^{\text{’}}$) are the corresponding error in the two coordinates system.

From Section 2.1 and Section 2.2, the angle measurement error of the rotary-laser transmitter directly affects the establishment of the equation of the laser plane. The impact of this error is analyzed in Equation (20). It is gotten by substituting angle measurement error into Equation (6).

(20) $$∆n_{mi}=\left[\begin{array}{c} ∆a_{mi}\\ ∆b_{mi}\\ ∆c_{mi} \end{array}\right]=\left\{\begin{array}{l} \left[\begin{array}{ccc} \cos(\theta_{mi}+∆\theta_{mi}) & -\sin(\theta_{mi}+∆\theta_{mi}) & 0\\ \sin(\theta_{mi}+∆\theta_{mi}) & \cos(\theta_{mi}+∆\theta_{mi}) & 0\\ 0 & 0 & 1 \end{array}\right]\\ -\left[\begin{array}{ccc} \cos\theta_{mi} & -\sin\theta_{mi} & 0\\ \sin\theta_{mi} & \cos\theta_{mi} & 0\\ 0 & 0 & 1 \end{array}\right] \end{array}\right\}n_{m0}(m=1,2)$$

where $n_{m0}$ is the light plane coefficient at initial position, $∆n_{mi}$ is coefficient error of light plane caused by angle measurement error, $\theta_{mi}$ is the rotary angle of m light plane, and $∆\theta_{mi}$ is angle error caused by the transmitter.

**Figure 6:**
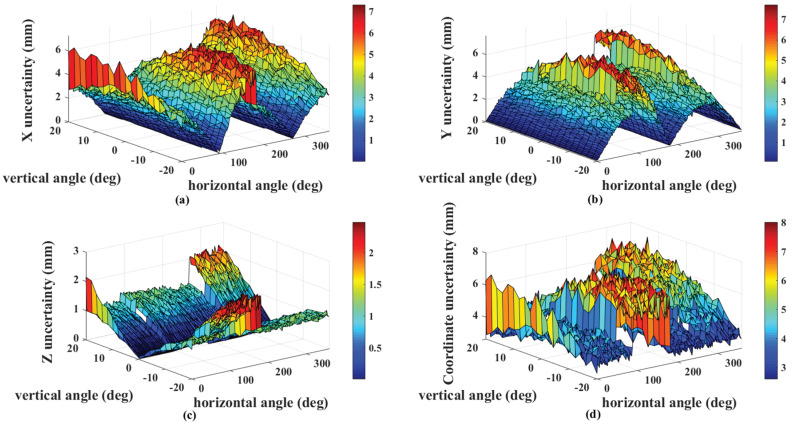
Error simulation in radius of 10 m: (**a**) the uncertainty of the X-axis error in space; (**b**) the uncertainty of Y-axis error; (**c**) the Z-axis uncertainty; (**d**) the uncertainty of combination error from three axes.

The target is calibrated by laser tracker, which brings little error—far less than the guidance error caused by angle measurement error. Therefore, based on this theoretical analysis, an error simulation for angle measurement error has been conducted as follows. The coordinate system of the transmitter is regarded as the global coordinate system in this simulation. The retroreflector on the target moves on a curved surface 10 m away from the transmitter and tracker, with the horizontal angle ranging from 0 to 360 degrees and vertical angle ranging from −20 to 20 degrees. We rotate the probe so that the six receivers face the transmitter at each simulation position. We calculate the theoretical rotation and translation parameters. The horizontal angle increases from 0 to 360 degrees with steps of 2 degrees, while the vertical angle increases from −20 to 20 degrees with steps of 2 degrees. One arc-second angle uncertainty was added for each receiver, and we calculated the measured coordinates of the probe in the global coordinate frame. For each simulation position, we calculate the measured coordinates 100 times, and the standard deviations are considered as the coordinate uncertainty, which is shown in Figure 6. From simulation experiment results, the maximal guidance error is 6.08 mm, and the maximal angle guidance error is less than 0.035 degrees—far less than the searching angle of laser tracker

## 4. Experiments and Discussions

### 4.1. Experimental Conditions

**Figure 7:**
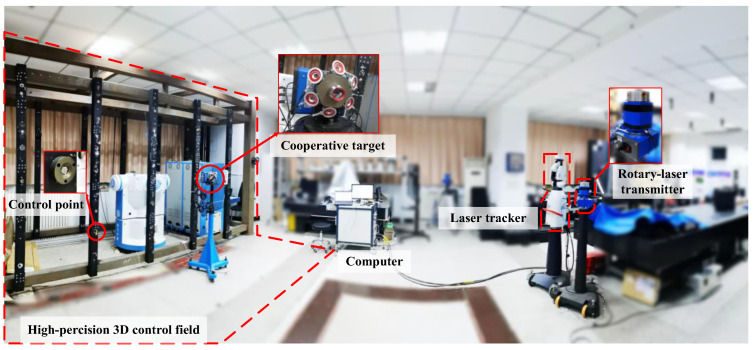
The layout of the devices, cooperative target, and the high-precision control field.

In order to verify the guidance method mentioned above, we established an experimental environment as shown in Figure 7. A new generation of high-precision wMPS transmitter is used to realize the rotary-laser scanning, and the repeating scanning angle measurement accuracy of the transmitting station is 1 arc second. The laser tracker we used is Leica AT901, with measurement accuracy of 15 μm/m ± 6 μm/m in the total measurement range.

As seen in Figure 7, a compact measurement target was designed and used in the experiment. The target was directly calibrated using laser tracker, and coordinates of receivers and retroreflector on TCS are shown in Table 1, where the point 1–6 are receivers in TCS and point 7 is the retroreflector in TCS.

As the guiding principle introduced in Section 3.3, we established a control field within a range of 1.5 m × 1.5 m × 1.5 m at the distance of 4 m in front of the laser tracker, and calibrated transmitter by using 12 control points. The calibrated external parameters of $R_{SL}$ and $T_{SL}$ are listed below.

$$\begin{array}{c} R_{SL}=\left[\begin{array}{rrr} 0.310197 & -0.95033 & -0.02553\\ 0.950445 & 0.310598 & -0.01353\\ 0.020787 & -0.02007 & 0.999582 \end{array}\right]\\ T_{SL}=\left[-17.8567\;-584.0616\;44.1895\right] \end{array}$$

### 4.2. Site-Guided Experiment

The target was fixed with the receivers and placed in the measurement workspace to record the scanning time of the transmitter continuously. The average value of scanning time was generated into a log with n-times periods; then the coordinate was calculated by MATLAB which would be sent to the controlling software of wMPS, which was developed by Visual Studio. The coordinates of retroreflectors in LCS would be recorded, and the program developed by EMSCON would finally guidance the laser tracker to target. The overall layout of the experimental equipment is shown in Figure 7, and the recorded scanning times from receivers are listed in Table 2.

In Table 2, the $\theta_{1i}$ and $\theta_{2i}$ are rotated angles of the two scanning laser planes from the initial position to the *i*-th receiver, respectively. These angles are calculated by Equation (5) based on scanning time recorded by receivers. Through the method introduced in Section 3.3, the rotation matrix and translation matrix between SCS and TCS, $R_{TR}$ and $T_{TR}$, are calculated:

$$\begin{array}{c} R_{TS}=\left[\begin{array}{rrr} 0.874538 & -0.48036 & -0.06665\\ 0.031854 & 0.194045 & -0.98048\\ 0.48391 & 0.855339 & 0.185001 \end{array}\right]\\ T_{TS}=\left[-138.750\;-3651.352\;102.147\right] \end{array}$$

Coordinates of retroreflector from guidance system are (3425.063, −1859.826, 275.932) mm, while the exact values are (3423.445, 1858.897, 275.814) mm. Guidance error is less than (1.62, 0.93, 0.12) mm. Positioning standard deviations of the 20 guided experiments are (0.24, 0.13, 0.09) mm. Result of single-point positioning experiment shows the feasibility of the automatic guidance method based on rotary-laser scanning angle measurement. In the following experiment, the measurement accuracy and the guidance ability in horizontal full angles are verified.

### 4.3. 360° Guidance Experiment

**Figure 8:**
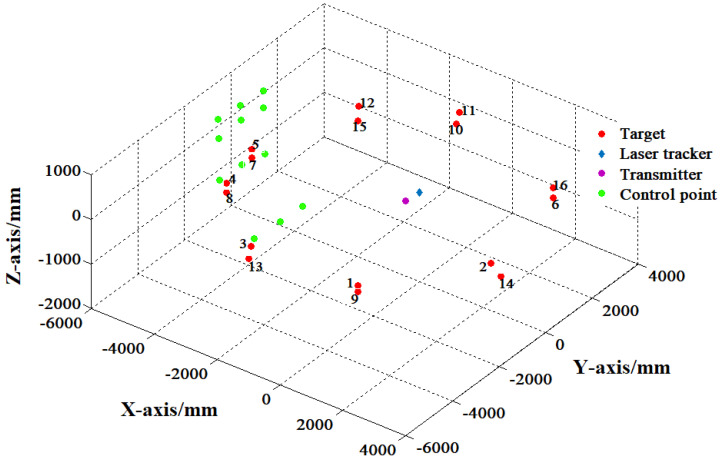
Layout of the 360° guidance experiment.

**Figure 9:**
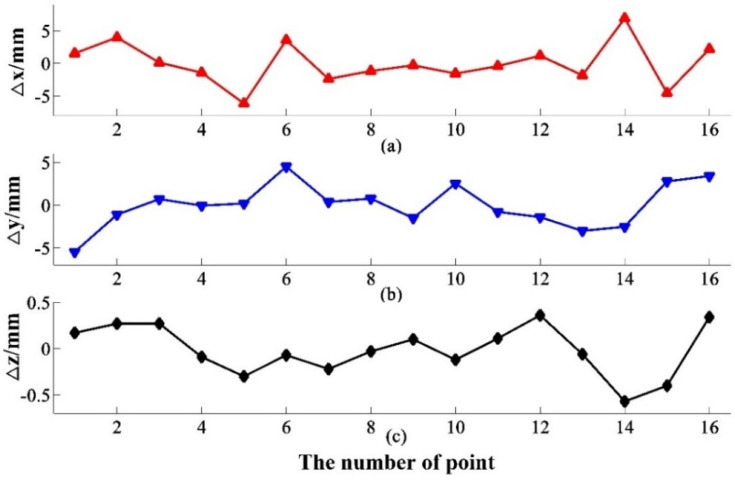
Guidance error of trajectory experiments in 360° field: (**a**) ∆x is x-coordinate error; (**b**) ∆y is y-coordinate error; (**c**) ∆z is z-coordinate error.

**Figure 10:**
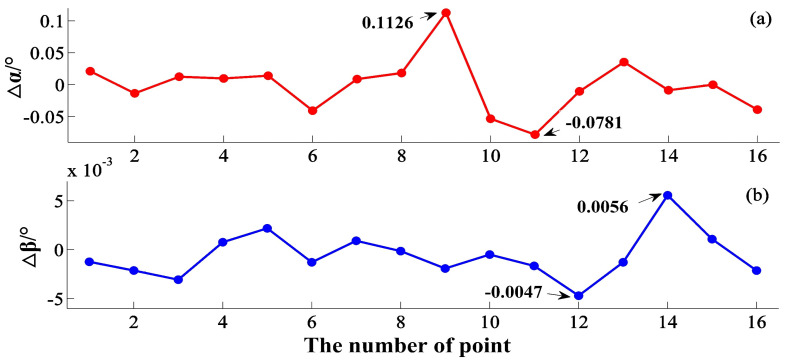
Guidance angle error of trajectory experiments in 360° field: (**a**) ∆α is the horizontal angle error; (**b**) ∆β is the vertical angle error.

In order to verify the system’s tracking and guiding ability, trajectory experiments are arranged here to verify the ability of 360° coverage. As shown in Figure 8, the measured points are distributed in the plane area about 8.5 m × 8.5 m, and the laser tracker and the transmitter are placed in the centre of the guidance area, about 3 m~5 m away from each measuring point. Experiment results are depicted in Table 3. From Figure 9, the 360° guidance error is less than 6.89 mm, and the maximal guidance angle error within the distance of 5.07 m, as shown in Figure 10, is 0.1126 degrees, which is far less than the laser trackers’ searching angle, ±5 degrees. The repeatability of the 20 measurement results with little difference is less than 0.24 mm. Most laser trackers support reconnection of interrupted laser beam in a 10-degree field. The measurement accuracy of the guidance system proposed in this paper could satisfy the guidance requirement. For the laser tracker without the function of reconnection of the interrupted laser beam, the radius of the retroreflector entrance is about 16.5 mm, which also meets the guidance requirement.

### 4.4. Discussion

Concerning the automatic guidance result proposed in this paper, there are several issues to be discussed here:

1. The automatic guidance experiment results show that the maximum alignment coordinate error is 6.89 mm and that the angle error is less than 0.1126 degrees. Considering that most laser trackers support reconnection of interrupted laser beam in ±5 degrees field, the measurement accuracy of the guidance system proposed in this paper is far beyond the search field of laser tracker and could satisfy the guidance requirement fully.
2. The guidance method currently used by laser trackers is the vision guidance method, which suffers from the limitation that the view field can only cover a 10-degree view field, and the searching time is about seconds. The 360° coverage based on visual measurement technology could be applied after secondary development, but the conduction will be time-consuming, while the searching time of the method based on rotary-laser scanning technology is tens of arc-seconds. The method proposed in this paper could enlarge the searching field from tens of degrees to horizontal full angles, shorten the searching time from seconds to arc-seconds, help laser tracker get rid of the dependence on manual participation, and greatly improves the measurement efficiency.
3. Several points here have not been the best and could be improved in the future. For example, more errors in practice have not been considered in this paper, such as target vibration and instrument stability, which caused the differences between experimental results and simulations. Under the conditions of limited laboratory space, we conducted the 360° guidance experiment within a range of 3–5 m, which is not enough to verify the change of guidance error as the measurement distance increases. The guidance process of the proposed method needs communications during several device; therefore, the process control software requires more manual control instructions, and maybe a simple process could be provided in further work.

## 5. Conclusions

Laser trackers are now a standard-issue metrology tool for in-place inspection of large parts and assemblies in both the automotive and aerospace industries. However, the existing guidance methods for laser trackers only support the reconnection of interrupted laser beam in view field of ±5 degrees with limited measurement efficiency. This paper presents an automatic guidance system for the laser tracker based on the rotary-laser scanning technology, where the transmitter of wMPS is regarded as the rotary-laser scanning device. A compact target was designed to cooperate with the coordinates of retroreflector and receivers. The automatic guidance principle was introduced in detail, including the angle measurement principle of wMPS, calibration method of the guidance system, and the integration of retroreflector and receivers. The guidance system error analysis was conducted, and the results were shown in the error simulation experiment. The guidance experiments verified the feasibility, great robustness, and the ability of 360° coverage of this automatic guidance method. Experiment results reveal that the method proposed in this paper could shorten the searching time in a 360° field. This method improved the measurement efficiency greatly with the guidance repeatability less than 0.24 mm and guidance accuracy better than 6.89 mm in an area about 8.5 m × 8.5 m, which satisfies the requirement of laser tracker guidance absolutely.

With regard to the method proposed in this paper, some more investigation and detailed work could be done, such as a method for integration of programs and accuracy improvement. In addition, the potential application of this proposed method in guiding other high-precision measuring equipment or combined with distance measurement technology to replace the target would also be studied in the future.

## Figures and Tables

**Table 1:** Coordinates of receivers and retroreflector in TCS.

Num	x/mm	y/mm	z/mm	Num	x/mm	y/mm	z/mm
1	0.000	0.000	0.000	5	80.714	46.594	0.037
2	53.824	0.000	0.000	6	−26.903	46.583	−0.083
3	53.773	93.190	0.000	7	26.928	46.656	31.993
4	−0.021	93.188	−0.058				

**Table 2:** The scan time of the two laser planes from initial position to six receivers.

Num(*i*)	$t_{1}\left(ms\right)$	$t_{2}\left(ms\right)\;$	*T(ms)*	$\theta_{1i}$	$\theta_{2i}$
1	9.196162	17.74847	35.28616	93°49′19.2″	181°4′30.7″
2	9.311836	17.78424	35.28616	95°0′7.7″	181°26′24.6″
3	9.394737	17.71765	35.28616	95°50′52.5″	180°45′38.8″
4	9.364238	17.61358	35.28616	95°32′12.3″	179°1′56.5″
5	9.249788	17.57488	35.28616	94°22′8.8″	179°8′15.2″
6	9.166832	17.64194	35.28616	93°31′22.0″	179°59′18.0″

**Table 3:** Coordinate value and deviation of 360° guidance experiment.

	Laser Tracker			wMPS			Error		
Num	x/mm	y/mm	z/mm	x/mm	y/mm	z/mm	dx/mm	dy/mm	dz/mm
1	1402.46	−4539.43	56.31	1403.99	−4544.9	56.48	1.53	−5.47	0.17
2	3590.33	−1769.99	119.61	3594.3	−1771.1	119.88	3.97	−1.11	0.27
3	−2020.88	−4521.25	−33.49	−2020.76	−4520.54	−33.22	0.12	0.71	0.27
4	−4343.63	−2426.69	−93.19	−4345.05	−2426.74	−93.28	−1.42	−0.05	−0.09
5	−4769.98	−762.45	−92.19	−4776.12	−762.26	−92.49	−6.14	0.19	−0.3
6	2786.34	1982.79	−91.08	2789.9	1987.3	−91.15	3.56	4.51	−0.07
7	−4815.45	−700.17	−322.65	−4817.83	−699.78	−322.88	−2.38	0.38	−0.22
8	−4374.47	−2400.61	−323.52	−4375.63	−2399.85	−323.55	−1.16	0.77	−0.03
9	1265.46	−4372.4	−173.5	1265.18	−4373.92	−173.4	−0.29	−1.52	0.1
10	−1411.98	3487.66	−195.31	−1413.54	3490.2	−195.43	−1.56	2.54	−0.12
11	−1350.83	3528.55	61.29	−1351.24	3527.78	61.39	−0.42	−0.78	0.11
12	−3662.02	2337.28	−9.82	−3660.85	2335.87	−9.46	1.17	−1.4	0.36
13	−1990.06	−4649.43	−251.65	−1991.88	−4652.45	−251.71	−1.82	−3.01	−0.06
14	3880.2	−1752.35	−90.53	3887.08	−1754.87	−91.1	6.89	−2.52	−0.57
15	−3579.92	2193.41	−245.13	−3584.46	2196.19	−245.52	−4.54	2.78	−0.4
16	2858.32	1900.46	186.34	2860.52	1903.87	186.67	2.2	3.41	0.34
